# Case report: Preventive infusion of donor-derived CD7 chimeric antigen receptor T cells after allogeneic hematopoietic stem cell transplantation

**DOI:** 10.3389/fimmu.2024.1381308

**Published:** 2024-04-30

**Authors:** Ying Jiang, Dan Feng, Jun Zhu, Daolin Wei, Chuxian Zhao, Huixia Liu, Shan Shao, Chun Wang

**Affiliations:** Department of Hematology, Shanghai Zhaxin Traditional Chinese and Western Medicine Hospital, Shanghai, China

**Keywords:** HSCT, CD7 CAR-T, T-ALL/LBL, preventive infusion, complete remission

## Abstract

Chimeric antigen receptor T cells (CAR T) targeting CD7 for T-cell acute lymphoblastic leukemia/lymphoma (T-ALL/LBL) showed promising efficacy and safety in some clinical trials. However, most of them were bridged with allogeneic hematopoietic stem cell transplantation (allo-HSCT). We described successful treatment with preventive donor-derived anti-CD7 CAR-T therapy in a case of refractory T lymphoblastic lymphoma following allo-HSCT, who could not receive autologous anti-CD7 CAR-T products due to the low-quality of T lymphocytes. To date, the patient’s complete remission has persisted for 20 months after HSCT.

## Introduction

T-ALL/LBL is an invasive hematological malignancy caused by malignant transformation of immature T-cell progenitors (1). Only 20%–40% of T-ALL/LBL patients can be alleviated by multidrug chemotherapy (2). Allo-HSCT is recommended in patients with T-ALL/LBL in complete remission (CR). In the cases who did not achieve CR, although a variety of salvage chemotherapy regimens may be used, response rates are low. Overall survival from refractory T-ALL/LBL is poor (<10%) (3). Therefore, patients with refractory T-ALL/LBL have limited therapeutic option and a poor prognosis (4).

CD7, a transmembrane glycoprotein member of the immunoglobulin superfamily, has been widely evaluated as an antigen target, given its high expression on malignant T cells (5). In addition, clinical data have shown that CD7 is continuously expressed on cancerous T cells at higher levels than that on T cells from normal donors and is therefore considered an attractive target for CAR T-cell therapy (1). In addition, patients achieved deep remission after the CD7 CAR T-cell therapy despite of unfavorable genetics and resistance to all the available salvage treatments were reported (6). However, most of CD7 CAR T were bridged with allogeneic hematopoietic stem cell transplantation (allo-HSCT) to avoid such issues as T-cell aplasia, graft-versus-host disease (GVHD), and life-threatening infection (2).

CD7 CAR T cells were continually detected in the patients without HSCT consideration and disappeared later on in the patients with HSCT consideration (2, 7). Whether combined HSCT with sustained CD7 CAR T cells can provide better safety and efficacy or not is still unknown. We describe the use of preventive donor-derived CD7 CAR T after allo-HSCT a patient with refractory T lymphoblastic lymphoma.. She has achieved continuous complete remission and long-term disease-free survival with manageable adverse events.

## Case description

A 16-year-old girl had received a diagnosis of T lymphoblastic lymphoma based on clinical presentation and invasive mediastinal biopsy, with negative results in bone marrow aspiration at 13 years of age. During an emergency department visit for 10-day history of persistent cough, chest tightness, and abdominal pain at month 19 before HSCT, the patient was noted to have masses in the anterior mediastinum and left hepatic lobe (maximum: 136mm × 127mm ×84mm) with pericardial and right pleural effusion scanned by computed tomography. After resection of the anterior mediastinal tumor, a tumor measuring approximately 15cm × 15cm ×12 cm and part of the pericardium were removed. It is considered T-cell lymphoblastic lymphoma/leukemia with mediastinal mass and in the pleural fluid; CD10, CD3, and CD7 were expressed, and Ki-67, CD79a, CD99, Bcl6, Bcl2, TdT, CD1a, and CD38 were partly expressed.

A total of 11 cycles of chemotherapy including VDLP, CAM, HR1/2/3, and VDL regimens accompanied by repeated intrathecal therapy were performed from 18 months to 4 months before HSCT. The best and post-therapy responses were partial remission (PR) and progressive disease (PD) assessed by F-18 fluorodeoxyglucose positron emission tomography computed tomography (PET/CT) at month 15 and 3 before HSCT.

**Figure 1:**
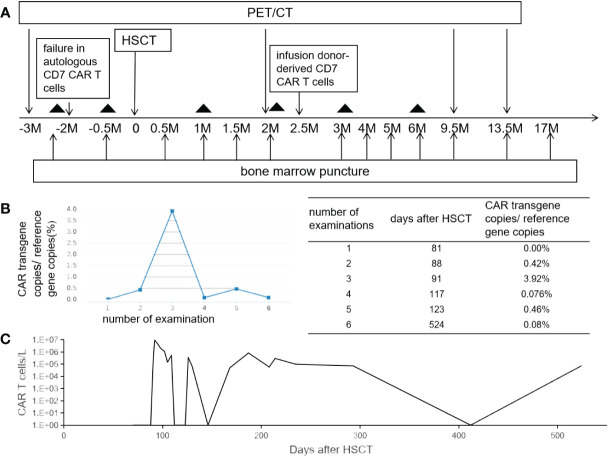
The schema and CAR T-cell persistence in peripheral blood. Panel **(A)** shows treatment schema. The black triangle indicates the date of lumber puncture. Panels **(B, C)** show the kinetics of CAR vector transgene copies measured by quantitative PCR and CAR T cells in the peripheral blood measured by flow cytometry.

Then, the patient was admitted in our hospital and planned for autologous CD7 CAR T-cell infusion. The CD7 CAR T-cell manufacture and detection were performed as previously reported (8). However, after the lymphocyte collection and lymphodepleting chemotherapy (fludarabine 50 mg d1–3 and cyclophosphamide 500 mg d1–3), we were informed of the failure of harvesting the autologous CD7 CAR T-cell. Therefore, allogeneic hematopoietic stem cell transplantation followed by preventive infusion of allogenic CD7 CAR T-cell from the same donor were performed (**Figure 1A**). Stem cells of haploidentical donor were infused (patient’ sister, HLA typing 7/12, donor blood group A Rh-positive, recipient blood group O Rh-positive, CD34+ cell count of 13.03×10^6^/kg) with a condition regimen of TBI 10Gy, VP-16 15mg/kg/d for 2 days, CTX 50mg/kg/d for 2 days, and ATG 2.5mg/kg/d for 4 days from day −8, and graft-versus-host disease (GVHD) prophylaxis included tacrolimus (0.02mg/kg/d) from day −2 and methotrexate (MTX) at days +1, +3, and +6. Neutrophil and platelet engraftment occurred in day +14 and +12, and full donor chimerism of the bone marrow was observed at day +14. Complete remission (CR) was achieved at month 2 following HSCT evaluated by PET/CT.

**Figure 2:**
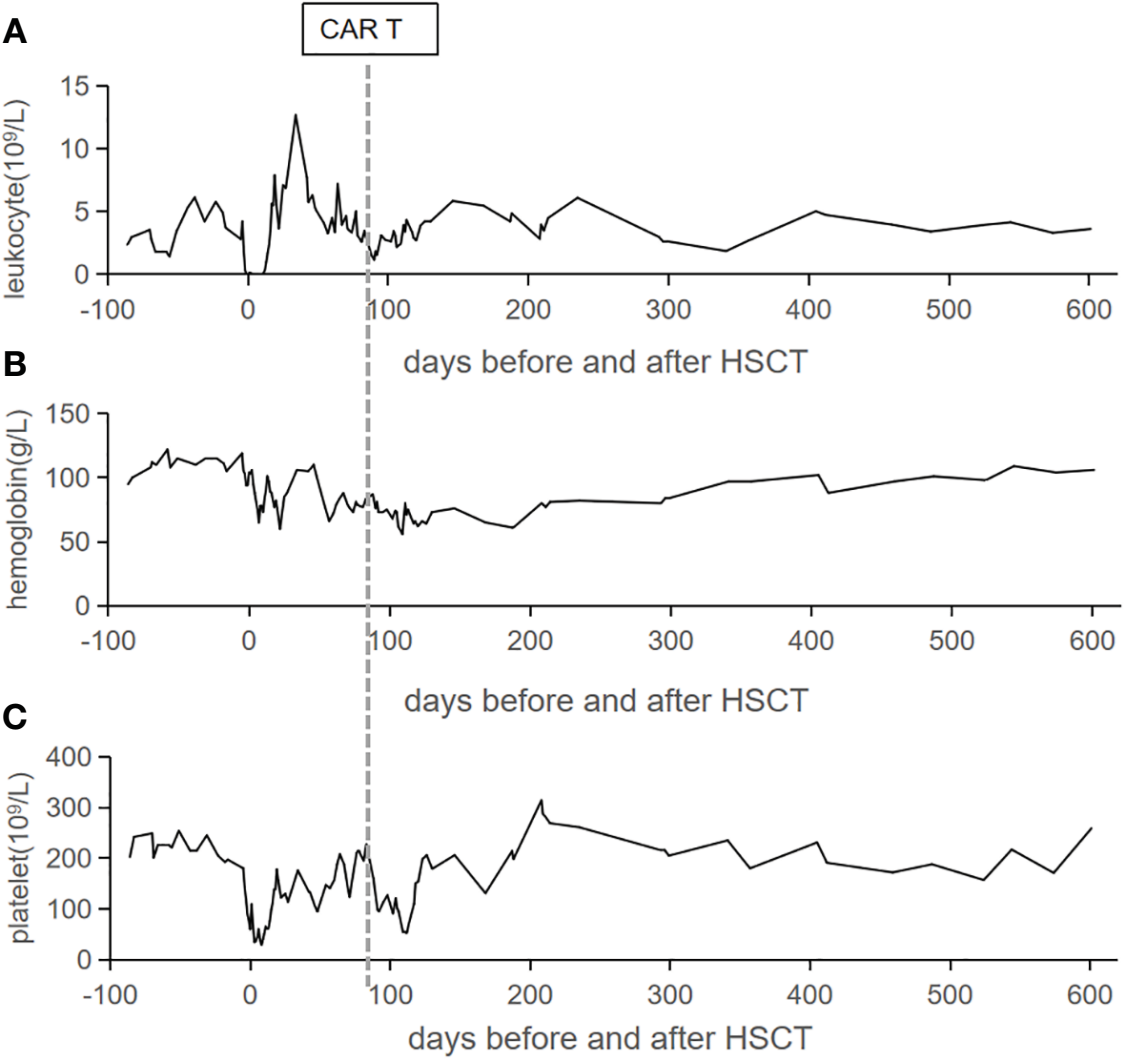
Peripheral blood cell count trends. The counts of leukocyte **(A)**, hemoglobin **(B)**, and platelet **(C)** in the peripheral blood are listed from admission to our hospital. The vertical line represents the date of CAR T cells infusion.

**Figure 3:**
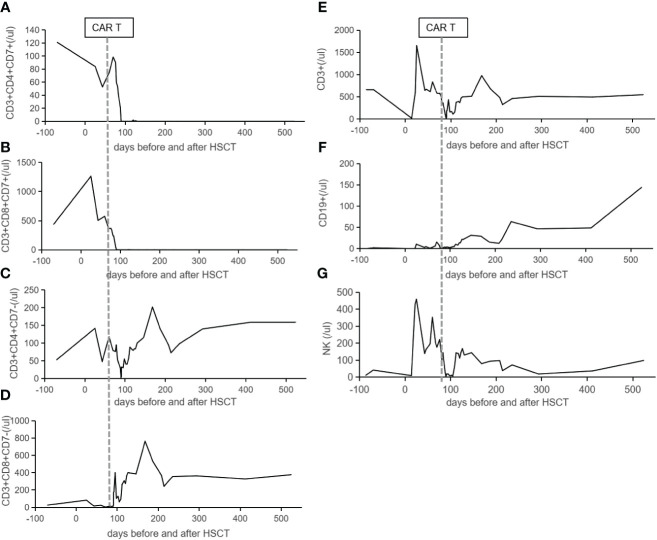
The lymphocytes reconstitution in the peripheral blood. CD7-positive cells of helper **(A)** and cytotoxic **(B)** T cells in the peripheral blood dropped to zero from day +17 following CAR T-cell infusion. Panels **(C, D)** show CD7-negative cells of helper and cytotoxic T cells in the peripheral blood expanded from day +17 following CAR T-cell infusion. The reconstitutions of CD3+ T cells **(E)**, CD19+ B cells **(F)**, and CD16+/CD56+ NK cells **(G)** are listed. The vertical lines indicate the date of CAR T cells infusion.

**Figure 4:**
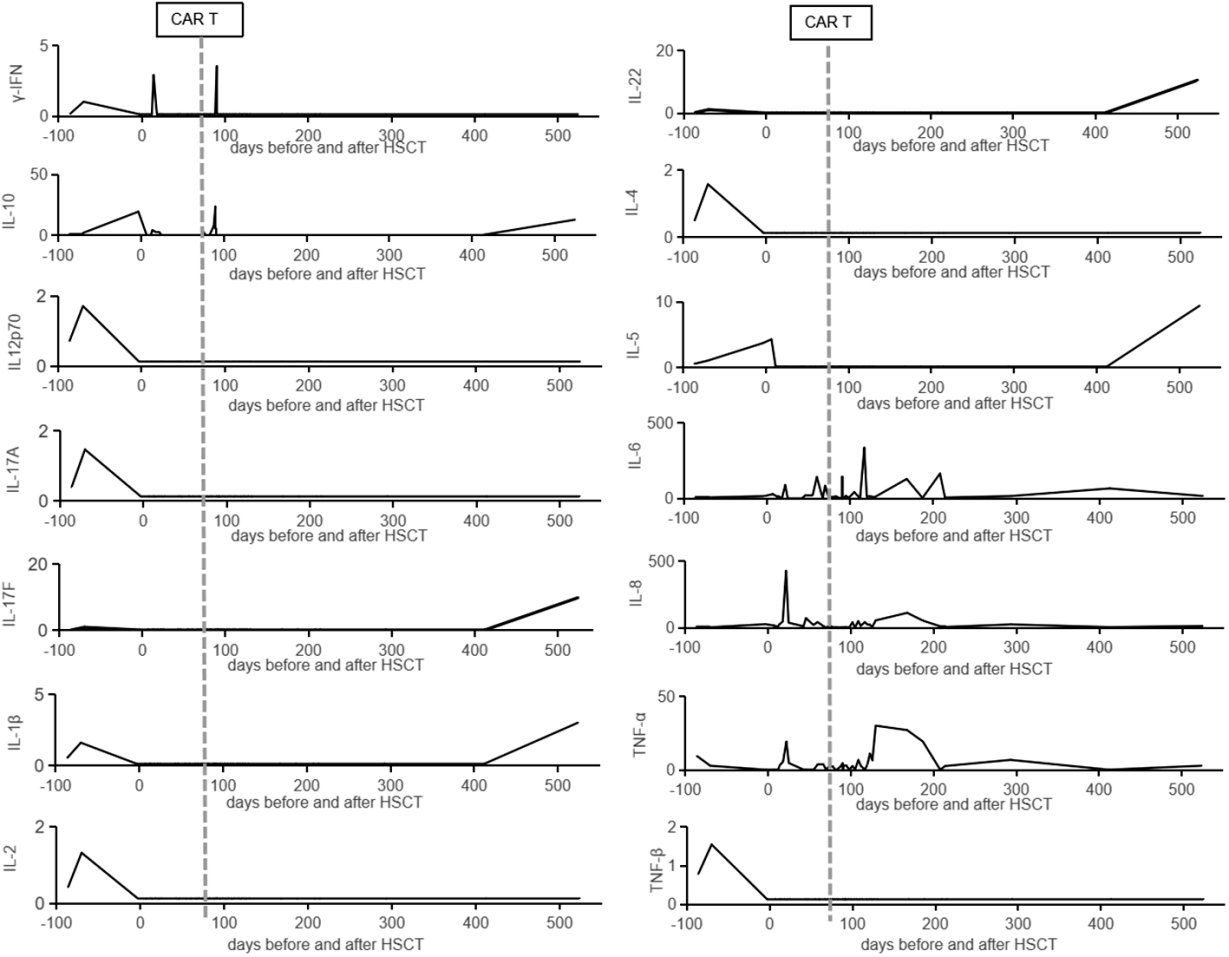
The cytokines data in the peripheral blood. The cytokines (γ-IFN, IL-10, IL-12p70, IL-17A, IL-17F, IL-1β, IL-2, IL-22, IL-4, IL-5, IL-6, IL-8, TNF-α, and TNF-β) in the peripheral blood are listed from admission to our hospital. The vertical lines represent the date of CAR T cells infusion.

**Figure 5:**
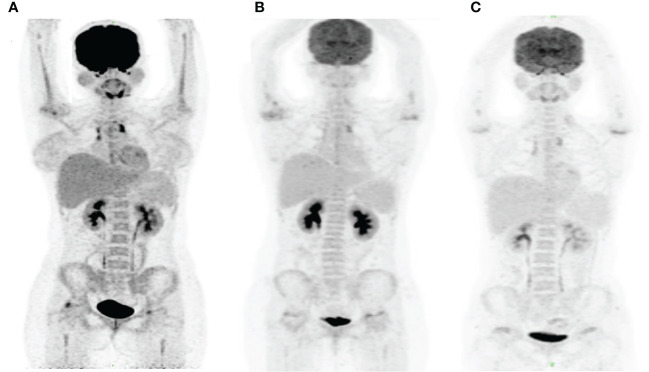
PET/CT images in this patient. The PET/CT images were obtained at month 3 before HSCT **(A)**, month 2 after HSCT **(B)** and month 13.5 after HSCT **(C)**. Panel **(A)** shows the increased tracer uptake in anterior superior mediastinum and new lesions of hypermetabolic focus in the bilateral femoral head and right humeral head. Panels **(B, C)** demonstrate a persistent complete remission after HSCT.

CD7 CAR T-cell (1×10^6^/kg) from the same donor was infused with oral tacrolimus 0.5mg every 12 h instead of lymphodepleting chemotherapy, and, at the same time, peripheral blood lymphocyte count was 0.78×10^9^/L. CAR T-cell in the peripheral blood (PB) demonstrated peak expansion at 19 days post-infusion with 9.1×10^7^/L and continued to be detectable post-infusion (**Figures 1B, C**). Pancytopenia developed post-infusion was alleviated after 2 months following CAR T-cell infusion (**Figure 2**). CD7-positive cells of helper and cytotoxic T cells in PB dropped to zero from day +17 (**Figures 3A, B**), and CD7-negative cells of helper and cytotoxic T cells in PB expanded from the same time (**Figures 3C, D**). Therefore, the gradual recoveries of CD3+ T cells, CD19+ B cells, and CD16+/CD56+ NK cells were all observed following CAR T-cell infusion (**Figures 3E–G**). The elevations of serum cytokines (IL-6, IL-8, IL-10, γ-IFN, and TNF-α) were observed after HSCT and CAR T cells infusion (**Figure 4**). So far, the patient remains in continued CR with full donor chimerism for 20 months after HSCT assessed intermittently using the bone marrow, CSF, and PET/CT (**Figures 1A**, **5**).

The patient presented with recurrent fever, and mild abdomen pain was diagnosed with intermittent cholecystitis at month 1, 2, 10, and 13 after HSCT, which was mitigated by intravenous antibiotic and eventually cured by laparoscopic cholecystectomy at month 13 after HSCT. Moreover, she had upper respiratory tract infection at month 6 post-transplant, which was relieved by 1 week course of antibiotic treatment. Grade 1 cytokine release syndrome (CRS) reaction was observed from day +14 and alleviated with oral tacrolimus 0.5mg every 12 h plus methylprednisolone 4mg per day. Immunosuppressants were gradually reduced and withdrawn at month 16 after HSCT. Then, liver and oral mucositis GVHD developed from month 17 after HSCT, accompanied by CD7 CAR T cells expansion again (7×10^5^/L), which were mitigated by resumption of oral tacrolimus 0.5mg twice daily.

## Discussion

We reported a successfully preventive donor-derived CD7 CAR T-cell therapy post-allo-HSCT in a patient with refractory T lymphoblastic lymphoma who had experienced 11 cycles of chemotherapy and failed to obtain autologous CAR T cells.

Refractory T-ALL/LBL is particularly difficult to treat and has dismal outcomes (3). Therefore, allo-HSCT often is regarded as the final salvage option, but the proportion of prolonged event-free survival post-transplant is quite low (2). Hamilton et al. conducted a multicenter retrospective cohort study involving 208 adult T-ALL/LBL patients undergoing SCT from 2000 to 2014 and disclosed that the 5-year OS was only 14% for r/r patients with a median follow-up of 38 months (9). In addition, Itonaga et al. retrospectively analyzed 35 patients with T-ALL who experienced progression or relapsed after a first allo-HSCT between 1997 and 2010 and found that the median survival time after relapse or progression was 6.2 months (10). Thus, HSCT alone is not the optimal method for this patient, and more effective treatment for her is urgently needed.

Refractory hematology malignancy currently remains challenging to treat, but recent clinical studies of CAR T in patients with B-cell acute lymphoblastic leukemia (B-ALL) and acute myeloid leukemia (AML) have shown encouraging results (11, 12). In particular, Pan et al. demonstrated that donor-derived CD7 CAR T cells exhibited efficient expansion and achieved a high complete remission rate with manageable safety profile (8). These studies provide promising allogenic CAR T for refractory patients without opportunity to have autologous CAR T treatment.

There was little CAR T activity that remained following transplantation in those patients who experienced donor CD7 CAR T therapy and then bridged to allo-HSCT. It was reported by Li et al. that CD7 CAR T cells were detected early at a median period of 60 days (range, 14–90) post-transplantation in 5 of 11 evaluable patients and disappeared later on (2). In addition, re-expansion of CAR T cells or depletion of CD7+ cells after allo-HSCT was not observed in another study conducted by Lu et al. (13). However, the persistence of CD7 CAR T in PB demonstrated sustained anti-tumor effect against CD7 flag cells. Tan et al. provided a 2-year follow-up in 12 participants with r/r T-ALL/LBL after allo-HSCT with CD7-directed CAR T cells originated from donors and showed the that median duration of CAR T-cell persistence at flow cytometry level in the 12 patients without SCT consolidation was 255 (30–682) days (7). Moreover, CD7 CAR T cells were still detectable in two patients before their CD7− relapses and undetectable in a patient before his CD7+ relapse (7). The patient turns out to have long-term disease-free survival (20 months), which was longer than the documented PFS of 11.0 (range, 6.9–12.5) months reported by Tan et al., which should be attributed to HSCT and the persistence of CAR T in PB observed by flow cytometry and PCR. Therefore, it is a probably favorable status that malignant cells are placed in the spotlight of CAR T cells post-HSCT. Put in another way, the combination therapy of HSCT and long persistence of CD7 CAR T cells in PB should be a potential approach to minimizing relapse.

A large proportion of patients who suffered from such adverse events (AE) as CRS, pancytopenia, GVHD, and infection was reported after donor-derived CAR T-cells infusion (7). All of non-relapse mortality of 12 patients without SCT consolidation occurred in four cases (30%) who had severe infections. However, the incidence of severe CRS and GVHD was no more than 10%. In this patient, it was attributed to proper use of immunosuppressants such as corticosteroid and tacrolimus therapy in that omitting the lymphodepleting chemotherapy and expansion of CD7 CAR T cells could be successfully achieved. Then, the balance between the use of immunosuppressants and harness CRS or GVHD was important in influencing prognostic significance. In addition, pancytopenia and infection were mild and manageable in this patient.

## Conclusion

Preventive donor-derived CD7 CAR T-cell therapy post allo-HSCT might be a valid treatment in refractory T-ALL/LBL patients with controllable complications. Long-term tacrolimus therapy appears to be a key factor in the treatment and prevention of CRS and GVHD. The inclusion of more patients and extended observation time is needed for further evaluation of this therapy.

## Data availability statement

The original contributions presented in the study are included in the article/supplementary material. Further inquiries can be directed to the corresponding author.

## Ethics statement

The study involving human was approved by Shanghai Zhaxin Traditional Chinese and Western Medicine Hospital ethics committee. The study was conducted in accordance with the local legislation and institutional requirements. Written informed consent for participation in this study was provided by the participants’ legal guardians/next of kin. Written informed consent was obtained from the individual(s), and minor(s)’ legal guardian/next of kin, for the publication of any potentially identifiable images or data included in this article.

## Author contributions

YJ: Formal analysis, Project administration, Writing – original draft, Writing – review & editing. DF: Project administration, Writing – review & editing. JZ: Writing – review & editing. DW: Writing – review & editing. CZ: Writing – review & editing. HL: Writing – review & editing. SS: Writing – review & editing. CW: Writing – review & editing.
